# Influence of extraction techniques on the efficiency of pomegranate (*Punica granatum* L.) peel extracts in oxidative stability of edible oils

**DOI:** 10.1002/fsn3.3244

**Published:** 2023-02-04

**Authors:** Samira Javani‐Seraji, Behnaz Bazargani‐Gilani, Narjes Aghajani

**Affiliations:** ^1^ Department of Food Hygiene and Quality Control, Faculty of Veterinary Science Bu‐Ali Sina University Hamedan Iran; ^2^ Department of Food Science and Technology, Bahar Faculty of Food Science and Technology Bu‐Ali Sina University Hamedan Iran

**Keywords:** antioxidant activity, edible oils, extraction method, oxidative stability, pomegranate peel extract

## Abstract

In this study, the effects of pomegranate (*Punica granatum* L.) peel extract (PPE) on the oxidative stability of soybean oil and ghee were investigated under heat conditions. Three extraction methods (immersion, ultrasound, and combined immersion‐ultrasound) with eight solvents (hot water, cold water, absolute methanol, methanol 50%, absolute ethanol, ethanol 50%, absolute acetone, and acetone 50%) were used for the evaluation of the extracts. Ethanolic extract in maceration method significantly (*p* ≤ .05) showed the highest DPPH radical scavenging activity (95.018%), reducing power (3.981), and total phenolic content (520 mg GAE/g) compared to the other samples. Then, the effects of PPE in various concentrations (200, 400, 600, and 800 ppm) were compared to the synthetic antioxidant (Butylated hydroxytoluene 200 ppm) in the oxidative stability of soybean oil under 65°C and ghee under 55°C for 24 days with 6‐day intervals, respectively. During storage period, all treatments showed a significant decrease (*p* ≤ .05) in peroxide value, thiobarbituric acid reactive substances, conjugated dienes value, polar compounds value, and acid value compared to the control. Except for the PPE 200 treatment, the other treatments exhibited superior efficiency to the synthetic antioxidant in a dose‐dependent manner in accelerated stored edible oils. Based on the sensory analyses (flavor, odor, color, and overall acceptability), PPE significantly (*p* ≤ .05) preserved the sensory features compared to the control group during the entire storage time. PPE 800 ppm was the most efficient treatment in all analyses, followed by PPE 600, 400, and 200 ppm, respectively. Finally, it was concluded that PPE can be introduced as a unique alternative to synthetic antioxidants in edible oils under heating conditions.

## Graphical abstract

It was concluded that pomegranate (*Punica granatum L.*) peel extract can be introduced as a unique alternative to synthetic antioxidants in edible oils under heating conditions.

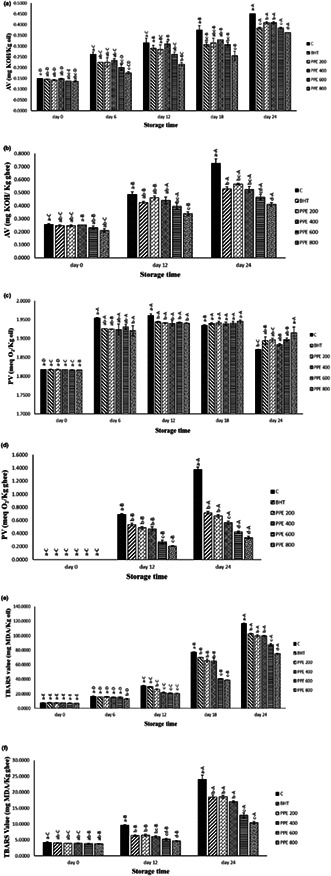

## 1 INTRODUCTION

Edible oils are among the most consumed food products in the world. Therefore, a large amount of this product is produced and consumed in the world every year. Edible oil producers widely use soybeans for the production and formulation of edible oils. Soybean oil is very susceptible to oxidation reaction during storage time or heat conditions that can be related to its high polyunsaturated fatty acids (PUFA; Rahmati et al., 2022; Tinello & Lante, 2020). Another type of the popular edible oil is ghee. Ghee is typically prepared by simmering butter of sheep or cow milk. Ghee can be used in cooking, frying, and dressing and creates a palatable and pleasant odor and taste in foods (Ahmad & Saleem, 2020). Cow ghee is rich in free fatty acids (FFA), phospholipids, glycerides, sterol esters, sterols, carotenoids, and fat‐soluble vitamins (Dhiman et al., 2022). During storage, the edible oils undergo oxidation reaction that can be correlated to the storage temperature and oxygen availability. The oxidation phenomenon of foods can lead to the loss of sensory features and customer‐friendliness that can be due to the generation of off‐flavor and off‐odor compounds (such as peroxides, aldehydes, polar compounds, and conjugated dienes). Therefore, the use of antioxidants for preserving their quality is inevitable (Chen et al., 2021). High safety and quality of edible oils can improve consumer health and prevent economic losses. Synthetic antioxidants are considered unpleasant by consumers and they prefer natural alternatives in foods. Herbal products, such as various extracts and essential oils with high health benefits, have been recently considered as food preservatives. Pomegranate with scientific name of *Punica granatum* L. from the Punicaceae family is a strategic commercial fruit crop that is widely cultivated in the Mediterranean, the Middle East, Asia, and North Africa. This product has many uses that lead to the production of a large amount of waste, including peel, seed, and pulp every year. Pomegranate peel extract is rich in bioactive phenolic and flavonoid compounds, such as anthocyanins, gallotannins, hydroxycinnamic acids, hydroxybenzoic acids, and hydrolyzable tannins, that is, ellagitannins, and gallagyl esters and complex polysaccharides (El‐Hadary & Taha, 2020; Rashid et al., 2022). Numerous investigations have reported the antimicrobial, antioxidant, and therapeutic properties of PPE (Kumar et al., 2022; Rashid et al., 2022; Trigo et al., 2020). In addition to the biological activities of pomegranate products, pomegranate derivatives can also be used as food colorants and flavor enhancers (More et al., 2022). Accordingly, this study aimed to measure the antioxidant activities of the produced PPE using immersion (maceration), ultrasound, and combined immersion‐ultrasound techniques with various solvents and evaluate their efficiency on chemical and sensory characteristics of soybean oil and ghee stored at 65 and 55°C for 24 days with 6‐day intervals in comparison with the chemical antioxidants, respectively.

## 2 MATERIALS AND METHODS

### 2.1 Materials

Tween 80, Butylated hydroxytoluene (BHT), Folin–Ciocalteu reagent, 2,2‐diphenyl‐1‐picrylhydrazyl (DPPH), and malondialdehyde (MDA) were obtained from Sigma‐Aldrich Chemie. Analytical grade acetone, ethanol, methanol, chloroform, butanol, hexane–isopropanol, ammonium thiocyanate, iron chloride (II) and (III), potassium hydroxide (KOH), phenolphthalein, disodium hydrogen phosphate (Na_2_HPO_4_), sodium dihydrogen phosphate (NaH_2_PO_4_), thiobarbituric acid (TBA), and 1,1,3,3‐tetraethoxypropane (TEP) were procured from Merck Company.

### 2.2 Preparation of PPE

Pomegranate peels were provided by a pomegranate processing plant in Hamedan. After drying the samples in environment temperature for 2 weeks, the samples were ground in the grinder (AR110O10, Moulinex) and mixed to the absolute and aqueous (50%) ethanol, methanol, acetone, and water (hot and cold) solvents with the ratio of 1:10 (powder/solvent) and then, were extracted by immersion (maceration), ultrasound, and ultrasound‐immersion methods. In the immersion procedure, the samples were shaken by the Earlene shaker (Fan Azma Gostar) at 250 rpm overnight at room temperature. For the immersion method by hot water, ground pomegranate peel (100 g) was refluxed with 1000 mL of the boiling distilled water (DW) for 1 h. In the ultrasound technique, a probe ultrasound (UP400ST, Hielscher) was considered under the power of 50, frequency of 20 kHz, and the temperature of 25°C for 30 min. In the ultrasound‐immersion method, first, the mixtures were subjected to ultrasound waves. Then, the obtained solutions were extracted by the immersion method. After filtering and concentrating the obtained solutions at 40°C by a rotary evaporator apparatus (Lab Tech), the solvent residue was evaporated by the vacuum oven (Fan Azma Gostar) at 50°C in all methods. The prepared extracts were stored in the laboratory freezer (Fan Azma Gostar) at −18°C for the next tests (Albu et al., 2004; Barkhordari & Bazargani‐Gilani, 2021; Pan et al., 2008).

### 2.3 Antioxidant activity of PPE

#### 2.3.1 DPPH radical scavenging activity

DPPH radical scavenging activity (RSA) of the studied extracts was determined based on the method of Blois (1958). A volume of 50 μL of the extracts was mixed with 2 mL of methanol DPPH (24 μg/mL) solution and mixed. The obtained solution was stored in dark at environment temperature for 60 min and the absorbance was read at 517 nm using a spectrophotometer (Thermo Spectronic, Helios Gamma). The DPPH RSA was calculated by the following equation:

$$\text{DPPH radical scavenging activity}\;\left(\%\right)=\frac{A_{\text{blank}}-A_{\text{sample}}}{A_{\text{blank}}}\times 100$$

where *A*_blank_ and *A*_sample_ are the absorbance of the blank and extracts, respectively. BHT solution (2 mg/mL) was used as the control.

#### 2.3.2 Reducing power

For reducing power measurement of the extracts, the method of Oyaizu (1986) was used. A volume of 1 mL of the extracts was mixed with 2.5 mL of the sodium phosphate buffer (0.2 M, pH 6.6) and 2.5 mL of potassium ferricyanide (1%). After 20 min incubation at 50°C, 2.5 mL of trichloroacetic acid (10%) was mixed with the resulting solution. Then, the mixture was centrifuged at 1789 *g* for 10 min. In the end, 2.5 mL of the mixture was added to 0.5 mL of ferric chloride (0.1%) and 2.5 mL of the DW and incubated for 10 min. Then, the absorbance was measured at 700 nm using a spectrophotometer (Thermo Spectronic, Helios Gamma), against blanks that contained all used reagents except for the extracts. Higher absorbance showed higher reducing power. The standard solution was prepared by BHT in the concentration of 2 mg/mL.

#### 2.3.3 Total phenolic content

Folin–Ciocalteu reagent test was used for total phenolic measurement. Briefly, 500 μL of the extracts was mixed with 2.25 mL of DW and then, 250 μL of the Folin–Ciocalteu reagent was added. The mixture was vortexed for 1 min and allowed to react for 5 min. Then, 2 mL of sodium carbonate (7.5%) was added. After incubation at room temperature for 120 min, the absorbance of each mixture was measured at 760 nm using a spectrophotometer (Thermo Spectronic, Helios Gamma). The same procedure was also used for a standard solution of gallic acid. Gallic acid solution was considered for drawing the standard curve. The total phenolic content was determined as milligram of gallic acid/gram of the sample (Machu et al., 2015).

### 2.4 Designing the groups

Fresh pure ghee and soybean oil without synthetic preservatives were purchased from oil processing plants in Hamedan. After dissolving the extract in the solvent (ethanol), the resulting solution was mixed with an emulsifier (Tween 80, 0.2%) and added to the ghee and soybean oil. The oils containing extract were then subjected to the mixing of 2 min through a homogenizer at 30,000 rpm (T 25 digital ULTRA‐TURRAX, IKA). The treatments were designed in six groups for each one, including: (1) PPE 0 ppm (negative control); (2) BHT 200 ppm (positive control); (3) PPE 200 ppm (ghee or soybean oil containing 200 ppm of PPE); (4) PPE 400 ppm (ghee or soybean oil containing 400 ppm of PPE); (5) PPE 600 ppm (ghee or soybean oil containing 600 ppm of PPE); and (6) PPE 800 ppm (ghee or soybean oil containing 800 ppm of PPE) of the strongest extract obtained in the antioxidant tests (absolute ethanolic PPE in immersion method) obtained in previous step. Tween 80 (0.2%) was used as an emulsifier. All of the designated groups were stored in an oven at 55 and 65 ± 1°C for 24 days and analyzed at 0, 6, 12, 18, and 24 days of the storage period, respectively (Tinello & Lante, 2020; Umeda & Jorge, 2021).

### 2.5 Chemical analysis of the treatments

#### 2.5.1 Conjugated diene value

The method of Saguy et al. (1996) was used for conjugated diene value (CDV) measurement of the samples. The oil sample was diluted (1:600 for the studied oils) with hexane (HPLC grade). An extinction coefficient of 29,000 mol/L (Privett & Plank, 1962) was utilized to quantify the concentration of conjugated dienes formed during oxidation. The absorbance of the diluted oils was read at 234 nm using a spectrophotometer (Thermo Spectronic, Helios Gamma) against hexane as blank.

#### 2.5.2 Polar compounds value

Nonpolar compounds were determined based on the method of Schulte (2004). A glass column (15 cm in length and 1 cm in diameter) packed with the hydrated silica gel (water/silica gel in the ratio of 5:95) was used for chromatography. The eluent was a mixture of isohexane and diisopropyl ether in the ratio of 85:15 (v/v). The oil sample (0.5 g) was loaded into the packed column and the nonpolar fraction was eluted by the eluent. After collecting the eluent, nonpolar compounds weight was determined. Then, polar compounds value (POV) was calculated by the following equation:

$$\mathrm{POV}=\frac{W_{s}-W_{n}}{W_{s}}\times 100$$

where *W*_s_ is the weight of the sample and *W*_n_ is the weight of the nonpolar compounds.

#### 2.5.3 Peroxide value

The peroxide value (PV) test measures the amount of generated peroxides in the samples. According to the method of the International Dairy Federation (Shantha & Decker, 1994), PV was measured. The sample (0.30 g) was mixed with 9.8 mL chloroform–methanol (3:7) in a glass tube. After adding 0.05 mL of the ammonium thiocyanate solution (10 mM), the sample was vortexed. Next, iron solution (II) (0.05 mL) was mixed with the resulting solution and agitated. Then, the samples were incubated at room temperature for 5 min. The absorbance of the mixture was read at 500 nm, using an Ultraviolet–visible spectrophotometer (Thermo Spectronic; Helios Gamma). PV of the treatments was calculated as milliequivalents (meq) of O_2_/kg of sample.

#### 2.5.4 Thiobarbituric acid reactive substances

Thiobarbituric acid reactive substances (TBARS) test determines the produced MDA in the oxidized oils (Baştürk et al., 2018). On the basis of the Iranian National Standards (10494‐2006), TBARS value was determined. Fifty milligrams of the sample was dissolved in butanol and adjusted to 25 mL in a volumetric flask. Then, 5 mL of the obtained solution was added to 5 mL of the TBA solution (0.02 M), and placed in a water bath at the boiling temperature for 120 min. After cooling, the absorbance was measured at 530 nm using an Ultraviolet–visible spectrophotometer (Thermo Spectronic; Helios Gamma) against the water blank. A standard curve was determined using 1,1,3,3‐tetraethoxypropane (TEP) and the TBARS value of the treatments was calculated as milligram of MDA/kilogram of the sample.

#### 2.5.5 Acid value

Acid value (AV) of the samples was measured according to the International Standard ISO (660‐2009). This index is defined as the milligram of potash (KOH) needed to neutralize the FFA present in 1 g of oils. Ten grams of the sample was mixed with 50 mL of ethanol/chloroform solvent. Then, the sample was titrated in the presence of a phenolphthalein reagent with 0.1 N KOH. AV was calculated as milligram of KOH/kilogram of the sample.

### 2.6 Sensory analysis

The undergraduate students (20–22 years old) of the Department of Food Hygiene and Quality Control were chosen for the sensory evaluation of the treatments. Fresh potato slices (with the same dimensions) were salted (2%) and fried in the studied soybean oil at 180 ± 2°C for 5 min. The fried slices were subjected to sensory evaluation by the panelists in disposable plates. The ghee samples were directly evaluated by the panelists. A 5‐point hedonic scale was used to analyze the flavor (1: Extremely nonpalatable, 5: Extremely palatable), odor (1: Extremely unacceptable/off‐odors, 5: Extremely pleasant), color (1: Extremely undesirable, 5: Extremely great), and overall acceptability (1: Extremely unacceptable, 5: Extremely pleasant) (Bazargani‐Gilani & Pajohi‐Alamoti, 2020; Ramos et al., 2012).

### 2.7 Statistical analysis

This study was cross‐sectional and experimental with controlled trial and replicated twice. All the tests were performed in triplicate for every repetition (*n* = 2 × 3). The collected data were statistically analyzed by SPSS (IBM SPSS statistics 21) software and considered as mean values ± standard deviations (SD). The analysis of variance (ANOVA) and Tukey test were used at the significance level of *p* ≤ .05 to compare the means.

## 3 RESULTS AND DISCUSSION

**FIGURE 1:**
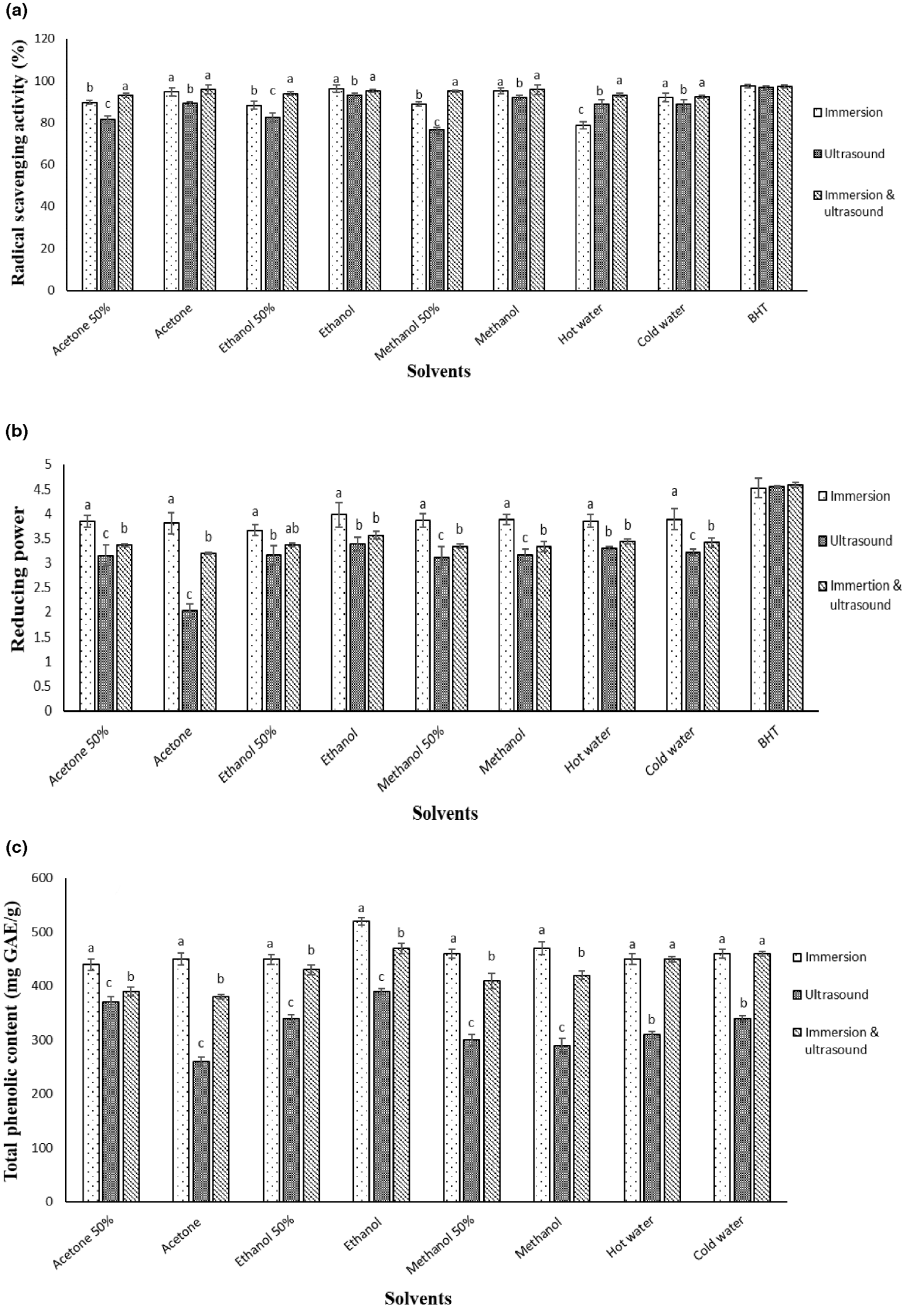
2,2′‐diphenyl‐1‐picrylhydrazyl (DPPH) radical scavenging activity (a), reducing power (b), and total phenolic content (c) of the prepared pomegranate peel extract (PPE) by different solvents and extraction methods. Different lowercases (a, b, and c) show a statistically significant difference (*p* ≤ .05).

**FIGURE 2:**
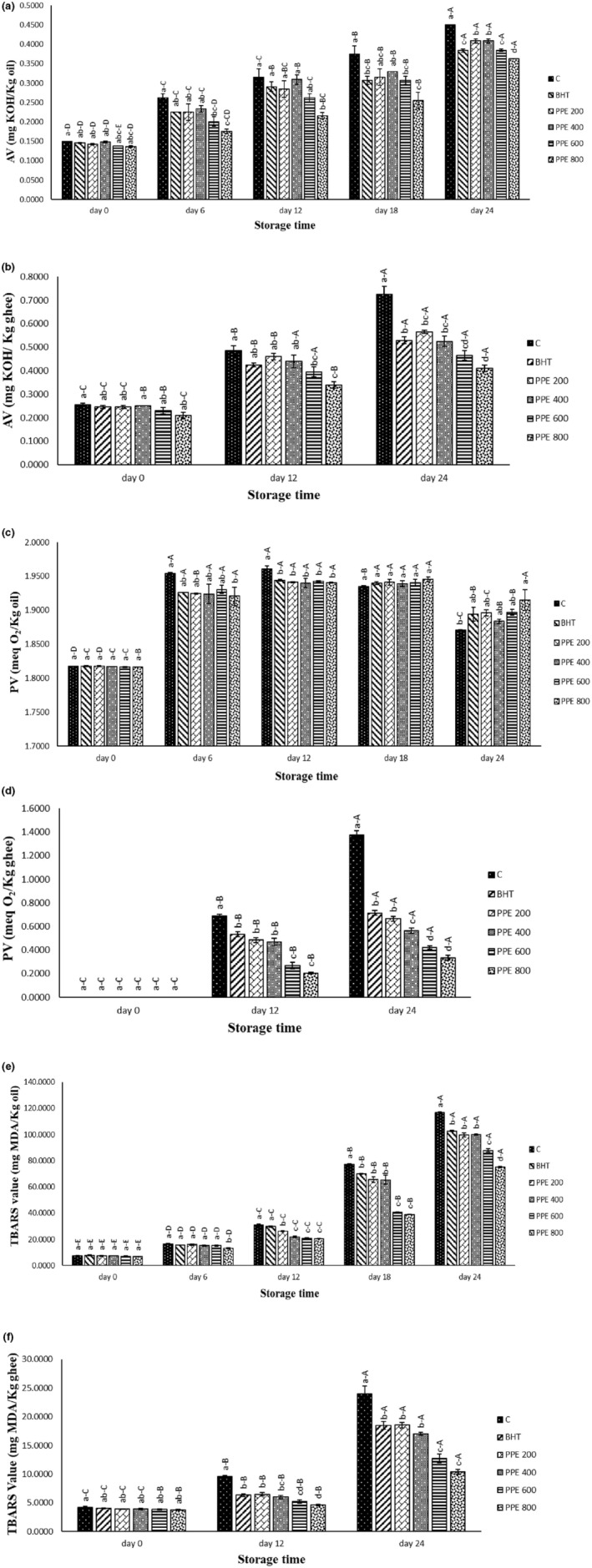
Acid value (AV) (a, b), peroxide value (PV) (c, d), and thiobarbituric acid reactive substances (TBARS) (e, f) of the treated soybean oils and ghee. Different letters within the same day (a, b, c, etc.) and the same treatment (A, B, C, etc.) show a statistically significant difference (*p* ≤ .05).

Figure 1a–c represents the antioxidant activity and total phenolic content of the studied extracts. The ability of the samples to scavenge DPPH radical was determined by DPPH test (Figure 1a). Reducing power assay measured the amount of reductant ingredients in the studied samples (Figure 2b). The total phenolic content of the samples is illustrated in Figure 1c. According to the obtained results, the highest RSA (96.308%), reducing power (3.981), and total phenolic content (520 mg GAE/g) belonged to the ethanolic PPE in immersion method among the others. Consistent with the other research, this result showed that the antioxidant activities of PPEs are directly related to the phenolic compounds of them so that the high release of phenolic substances could lead to the high antioxidant effects of the extracts (El‐Hadary & Taha, 2020; Kumar et al., 2022; More et al., 2022; Rashid et al., 2022; Trigo et al., 2020). The potent antioxidant activity of PPE can be related to the remarkable phenolic compounds, including phenolic acids, flavonoids, hydrolyzable tannins, proanthocyanidins, and anthocyanins. Phenolic acids are the main phenolic ingredients of PPE followed by hydrolyzable tannins, proanthocyanidins, and flavonoids, respectively. The major phenolic substance of the pomegranate peel is punicalagin, which causes its strong antioxidant activity (Selahvarzi et al., 2022). Also, it was found that the immersion and ultrasound‐immersion extraction methods were the successful techniques in the extraction of bioactive ingredients of PPE that can be due to the prolonged contact of the sample and solvent for solubilizing and then releasing the bioactive substances of the sample in the solvent. Furthermore, ethanolic extract showed the highest efficiency in the release of the antioxidant substances (phenolic compounds) of the PPE, while the other extracts exhibited similar performances in the same extraction method. Ethanol solvent, as GRAS (generally recognized as safe), is commonly used in the extraction process of various products. According to previous studies, ethanol showed high efficiency in solubilizing and releasing bioactive ingredients of natural products (Barkhordari & Bazargani‐Gilani, 2021; Esparvarini et al., 2022; Tavakkoli et al., 2020).

Figure 2a–f illustrates AV (Figure 2a,b), PV (Figure 2c,d), and TBARS value (Figure 2 e,f) of the treated soybean oil and ghee under accelerated conditions (accelerated storage aims to accelerate the rate of deterioration of the product without altering the mechanisms or order of changes seen in the product under normal storage conditions (Steele, 2004)). The AV index not only measures the amount of FFA in edible oils but also is a common factor in their identification. Hydrolysis of triglycerides by lipase enzyme increases the amount of FFA in the oils. The lipase enzyme can originate from animal or herbal tissues, whose fat or oil was extracted or as a result of contamination by other cells, such as microorganisms during processing or storage period (Wang et al., 2018). However, the hydrolysis reaction of triglycerides elevates the amount of AV, which indicates the poor quality of the oil. During poor processing and storage conditions of the oils, such as high relative humidity, heating, and tissue damage, the intensity of this reaction significantly increases. Since the released FFAs are very sensitive to the oxidation reaction, following the oil hydrolysis, oxidation is intensified (Rahmati et al., 2022). The oxidation reaction of edible oils is the most common phenomenon, which is accelerated by increasing their storage time. According to Figure 2a–f, there were no significant differences in PV, TBARS, and AV of all samples on the initial day of the storage period. By heating the samples next days, ascending trends were observed about three indexes. The samples containing PPE significantly (*p* ≤ .05) controlled these changes compared to the control group in a dose‐dependent manner during accelerated storage time. The hydrolysis reaction of the triglycerides occurs in the edible oils by increasing the storage time or under accelerated conditions. The formed FFA increase acid value of the oils and are simply oxidized during storage period. Peroxides and aldehydes (such as MDA) are primary and secondary products of the oxidation of the lipids (Rahmati et al., 2022). According to our findings, PPE could significantly (*p* ≤ .05) postpone hydrolysis followed by oxidation phenomena in the studied samples during accelerated storage time so that PPE 800 exhibited the highest efficiency among other samples and PPE 600, PPE 400, PPE 200, and BHT were in the next ranks, respectively. The lowest concentration of PPE (PPE 200) showed similar performance to the synthetic antioxidant (BHT) in most intervals and all indexes that can be related to the antioxidant activity of pomegranate peel. Previous studies demonstrated that pomegranate peel contains various polyphenol ingredients, such as phenolic acids, anthocyanin, hydrolyzable tannins, and flavonoids. Anthocyanins are the main bioactive (30%) substances in pomegranate peel that exhibit significant antioxidant activity effectively, preventing lipid oxidation (Kaderides et al., 2021; Selahvarzi et al., 2022). In general, PPE has the potential to improve the functional features of various food products (Selahvarzi et al., 2022; Trigo et al., 2020).

**FIGURE 3:**
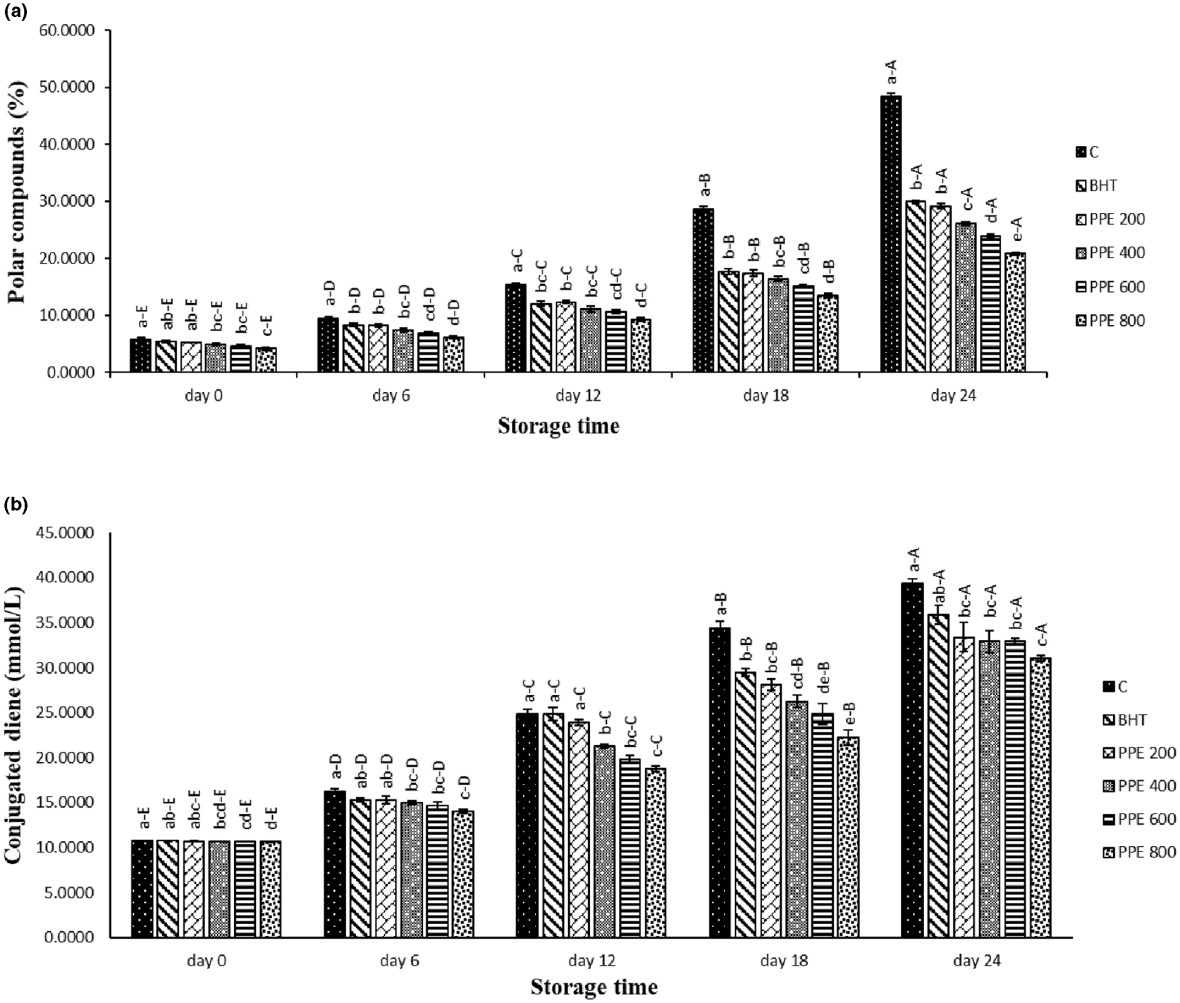
Polar compounds value (POV) (a) and conjugated diene (CDV) (b) of the treated oils. Different letters within the same day (a, b, c, etc.) and the same treatment (A, B, C, etc.) show a statistically significant difference (*p* ≤ .05).

Due to the high unsaturated fatty acid content in soybean oil, POV and CDV were measured only in the studied soybean oils. POV is dependent on linoleic acid (C18:2) and trans‐oleic acid (trans‐C18:1) content in the edible oils. The determination of total polar compounds is used as the main indicator in the evaluation of edible oil quality. The highest allowable value of total polar compounds is in the range of 24%–27% in edible oils (Chen et al., 2021). These oxidation products can damage human health and lead to a decrease of body weight and lipid content of tissues, liver, and blood. Therefore, the POV measurement in edible oils containing unsaturated fatty acids is essential in the evaluation of their quality (Xu et al., 2022). Figure 3a illustrates POV of the studied samples during storage period. There are no significant differences in POV (4.15%–5.75%) among the studied treatments on day 0 of the storage time. By increasing the accelerated storage period, the polar compounds of all samples increased significantly (*p* ≤ .05). PPE‐containing treatments showed significantly lower POV (*p* ≤ .05) compared to the control in a dose‐dependent manner. The lowest concentration of PPE (PPE 200) exhibited similar performance to the positive control (BHT 200 ppm) over the accelerated storage.

The presence of PUFA in edible oils leads to the production of conjugated dienes during accelerated oxidation time. No significant differences in CDV of the studied soybean oils were found on day 0 of the storage period (Figure 3b). According to the obtained findings, a significant ascending trend was found in all samples by increasing the accelerated storage period. Adding the PPE significantly decreased conjugated dienes of the samples over the accelerated oxidation period so that PPE 800 showed the best performance among the others and PPE 600, PPE 400, PPE 200, BHT, and control were in the next ranks, respectively. El‐Hadary and Taha (2020) reported that the highest amount of conjugated dienes of edible oils (sunflower, soybean, and corn) was in negative controls (without any antioxidants), indicating higher intensity of oxidation during the accelerated storage period, followed by methanolic PPE (100 ppm), positive control (containing synthetic antioxidant, TBHQ (tert‐Butylhydroquinone)‐200 ppm), PPE 200, PPE 400, and 600 ppm, respectively. In one study, higher significant oxidative stability of sunflower oil containing 800 ppm of PPE was reported in comparison with the synthetic antioxidant (BHT 200 ppm) (Ibrahium, 2010). In another study, PPE 250, 500, and 1000 ppm could delay corn oil deterioration during storage time, which was positively related to the concentration of PPE. The high amount of phenolic and flavonoid compounds of PPE have been reported to be responsible for this stability (Konsoula, 2016).

### 3.1 Sensory analysis

Tables 1 and 2 represent the sensory findings (taste, odor, color, and overall acceptability) of the studied samples during accelerated storage. By increasing the accelerated storage, the sensory scores of the fried potato slices in soybean oils and ghee decreased in all treatments. In agreement with the chemical analysis of the samples, PPE 800 treatment significantly (*p* ≤ .05) earned the highest scores in all sensory features over the storage period and PPE 600, PPE 400, PPE 200, and BHT treatments were in the next ranks, respectively. By delaying the chemical changes of the samples, PPE preserved the sensory attributes of the samples at an acceptable level (>3) until the end of the storage time. In other words, the generation of off‐flavor and off‐odor compounds (e.g., peroxides, aldehydes, polar compounds, and conjugated dienes) was significantly (*p* ≤ .05) decreased by PPE compared to the negative control group, leading to the high quality of the studied samples until the end of the accelerated storage period. Rahmati et al. (2022) reported that sumac fruit extract‐containing groups significantly (*p* ≤ .05) earned the highest scores of the sensory characteristics of the fried potatoes in the soybean oil under accelerated conditions compared to the control group and showed the same performance as the BHT‐containing treatment. Another study reported that the sunflower oil‐containing *Coriandrum sativum* essential oil (1200 ppm) showed desirable aroma and flavor over the accelerated storage period. Therefore, it could be considered as a customer‐friendly condiment (Wang et al., 2018). In previous research, it was observed that *Curcuma longa* leaves extract created significantly desirable sensory characteristics (flavor, color, oiliness, crispiness, and overall quality) in the fried potatoes in palm olein (*p* ≤ .05) compared to the positive (BHT) and negative control treatments during storage time (Nor et al., 2009). Previous studies reported that herbal products, such as Huai *Chrysanthemum morifolium* (Meng et al., 2021), black pepper and ginger (Chandran et al., 2016), and *Coriandrum sativum* L. (Wang et al., 2018) essential oils could not only increase the oxidative stability of edible oils but also improve the sensory features (taste, aroma, color, and overall quality) of them significantly (*p* ≤ .05) during accelerated storage period in a dose‐dependent manner. Therefore, these herbal extracts can be introduced as multipurpose food additives in edible oils with different health advantages.

**TABLE 1:** Changes in sensory features of fried potatoes in soybean oils during accelerated storage period.

Sensory attributes	Treatment	Storage period (days)		
		12	18	24
Taste	C	3.33 ± 0.51^b^	2.4 ± 0.54^c^	1.4 ± 0.54^b^
	BHT	3.66 ± 0.51^ab^	3.2 ± 0.44^bc^	1.6 ± 0.54^b^
	PPE 200	3.83 ± 0.4^ab^	3.2 ± 0.44^bc^	1.8 ± 0.44^b^
	PPE 400	4.5 ± 0.54^a^	3.8 ± 0.44^ab^	2.4 ± 0.54^ab^
	PPE 600	4.5 ± 0.54^a^	4.2 ± 0.44^a^	3.2 ± 0.44^a^
	PPE 800	4.5 ± 0.54^a^	4.4 ± 0.54^a^	3.4 ± 0.54^a^
Color	C	3.5 ± 0.54^b^	2.6 ± 0.54^b^	1.4 ± 0.54^d^
	BHT	4.16 ± 0.4^ab^	3.2 ± 0.44^ab^	2.0 ± 0.0^cd^
	PPE 200	4.16 ± 0.4^ab^	3.2 ± 0.44^ab^	2.2 ± 0.44^bcd^
	PPE 400	4.5 ± 0.54^a^	3.8 ± 0.44^a^	2.8 ± 0.44^abc^
	PPE 600	4.66 ± 0.51^a^	3.6 ± 0.54^a^	3.2 ± 0.83^ab^
	PPE 800	4.83 ± 0.4^a^	4.0 ± 0.0a	3.4 ± 0.54^a^
Odor	C	3.50 ± 0.54^b^	3.0 ± 0.70^b^	1.6 ± 0.54^b^
	BHT	3.83 ± 0.75^ab^	3.0 ± 0.70^b^	1.6 ± 0.54^b^
	PPE 200	3.83 ± 0.75^ab^	3.2 ± 0.44^ab^	2.0 ± 0.0^b^
	PPE 400	4.33 ± 0.81^ab^	3.54 ± 0.54^ab^	2.0 ± 0.0^b^
	PPE 600	4.66 ± 0.51^a^	3.8 ± 0.83^ab^	3.2 ± 0.44^a^
	PPE 800	4.83 ± 0.4^a^	4.4 ± 0.54^a^	3.8 ± 0.44^a^
	C	3.5 ± 0.54^b^	2.6 ± 0.54^c^	1.4 ± 0.54^d^
Overall acceptability	BHT	3.83 ± 0.4^ab^	3.0 ± 0.7^bc^	1.8 ± 0.44^cd^
	PPE 200	4.0 ± 0.63^ab^	3.4 ± 0.54^abc^	2.0 ± 0.0^cd^
	PPE 400	4.66 ± 0.51^a^	3.6 ± 0.54^abc^	2.4 ± 0.54^bc^
	PPE 600	4.5 ± 0.54^a^	3.8 ± 0.44^ab^	3.2 ± 0.44^ab^
	PPE 800	4.66 ± 0.51^a^	4.2 ± 0.44^a^	3.6 ± 0.54^a^

*Note*: Means within the same column (a, b, and c) with different letters are significantly different (*p* ≤ .05).

**TABLE 2:** Changes in sensory features of ghee during accelerated storage period.

Sensory attribute	Treatment	Storage period (days)		
		12	18	24
Overall acceptability	C	2.2 ± 0.44^b^	1.0 ± 0.54 ^e^	1.0 ± 0.0^b^
	BHT	2.8 ± 0.83^ab^	2.2 ± 0.44^d^	1.2 ± 0.44^b^
	PPE 200	2.8 ± 0.83^ab^	2.2 ± 0.44^d^	1.4 ± 0.54^b^
	PPE 400	2.6 ± 0.89^ab^	2.5 ± 0.44^c^	1.8 ± 0.83^ab^
	PPE 600	3.8 ± 0.83^ab^	3.0 ± 0.44^b^	2.2 ± 0.83^ab^
	PPE 800	4.0 ± 1.0^a^	3.4 ± 0.54^a^	2.8 ± 0.83^a^

*Note*: Means within the same column (a, b, and c) with different letters are significantly different (*p* ≤ .05).

## 4 CONCLUSION

The absolute ethanolic PPE showed the highest phenolic compounds, followed by the strongest antioxidant activities among the other extracts. In addition, this extract could significantly (*p* ≤ .05) increase the resistance of edible oils (soybean oil and ghee) against accelerated conditions compared to the control group. The most important conclusion from this study is that PPE showed similar efficiency to the synthetic antioxidant (BHT) in the preservation of soybean oil and ghee during the entire accelerated storage period in the same concentration (200 ppm). Therefore, considering that PPE is made from waste (pomegranate peel) with a natural base, it can be suggested as a strong, available, cost‐effective, and healthy alternative to the chemical preservatives in edible oils. Investigating other cooking methods with different temperatures on the quality of edible oils containing PPE can be the subject of future studies.

## CONFLICT OF INTEREST STATEMENT

No conflicts of interests were declared by the authors in this study.

## Data Availability

The data that support the findings of this study are available on request from the corresponding author.
